# Differences in phosphatemia and frequency of consumption of dietary sources of phosphorus in hemodialysis patients in southern and northern Brazil

**DOI:** 10.1590/2175-8239-JBN-2018-0063

**Published:** 2018-09-21

**Authors:** Fabiana Baggio Nerbass, Edcléia Regina Canzi, Renata dos Anjos Araujo, Dyane Corrêa, Rafaela Gonzaga dos Santos, Marcos Alexandre Vieira, Jyana Gomes Morais

**Affiliations:** 1 Fundação Pró-Rim, Joinville, SC, Brasil.; 2 Fundação Pró-Rim, Gurupi, TO, Brasil.; 3 Fundação Pró-Rim, Palmas, TO, Brasil.; 4 Fundação Pró-Rim, Jaraguá do Sul, SC, Brasil.

**Keywords:** Phosphorus, Diet, Renal Dialysis, Food Consumption, Fósforo, Dieta, Diálise Renal, Consumo de Alimentos

## Abstract

**Introduction:**

Hyperphosphatemia is associated with unfavorable outcomes, and the percentage of patients presenting with this condition in hemodialysis (HD) in kidney foundation units in the state of Santa Catarina (SC) is historically higher than that of patients in the state of Tocantins (TO).

**Objective:**

To assess the frequency of consumption of the main dietary sources of phosphorus and to compare them between the two states.

**Methodology:**

A cross-sectional study was carried out involving 123 patients, 66 of SC and 57 of TO: 52% were men, average age was 46.9 ± 15.7 years, and mean HD time 48 (57-71) months. A food frequency questionnaire (FFQ) with 33 items that are dietary sources of phosphorus was applied. A consumption score was calculated for sources of organic, inorganic, and total phosphorus, and the six-month average of phosphatemia was obtained.

**Results:**

The mean phosphatemia of SC patients was higher (6.2 ± 1.5 vs 4.7 ± 1.3 mg/dL, p <0001) than TO patients, as well as the prevalence of hyperphosphatemia (62% vs 28%; p <10001). In the total sample, the foods most frequently consumed were milk and beans. Comparing the frequency of consumption between the two states, a significant difference was found in 17 items. In TO, beef and beans were the foods most frequently consumed, and in SC, fourteen other items of the FFQ (pork, sausages, dairy products, etc.) were the most frequently consumed. Phosphatemia correlated with the frequency of consumption of inorganic phosphorus sources.

**Conclusion:**

the frequency of consumption of several items was different between the states, and this explains the differences in phosphatemia between the two regions.

## Resumo

**Introdução:**

A hiperfosfatemia está associada a desfechos desfavoráveis, e o percentual de pacientes que apresentam essa condição em unidades de hemodiálise (HD) de uma mesma instituição no estado de Santa Catarina (SC) é historicamente maior que o de pacientes do estado do Tocantins (TO).

**Objetivo:**

Conhecer a frequência do consumo das principais fontes dietéticas de fósforo e compará-las entre os dois estados.

**Metodologia:**

Estudo transversal do qual participaram 123 pacientes, 66 de SC e 57 do TO: 52% homens; idade=46,9±15,7 anos; tempo de HD=48 (57-71) meses. Foi aplicado um questionário de frequência alimentar (QFA) com 33 itens, fontes dietéticas de fósforo. Uma pontuação de consumo foi calculada para fontes de fósforo orgânico, inorgânico e total, e obtida a média semestral da fosfatemia.

**Resultados:**

A média da fosfatemia dos pacientes de SC foi maior (6,2±1,5 vs 4,7±1,3 mg/dl; p<0001), bem como a prevalência de hiperfosfatemia (62% vs 28%; p<0001). Na amostra total, os alimentos mais frequentemente consumidos foram leite e feijão. Comparando a frequência de consumo entre os dois estados, houve diferença significativa em 17 itens. Em TO, maior frequência de consumo de carne bovina e feijão, e em SC, de outros quatorze itens do QFA (carne suína, embutidos, laticínios, etc.). A fosfatemia se correlacionou com a pontuação de frequência de consumo de fontes de fósforo inorgânico.

**Conclusão:**

a frequência de consumo de vários itens foi diferente entre os estados, e esse achado pode ser uma das razões que explicam as disparidades na fosfatemia entre as duas regiões.

## INTRODUCTION

Hyperphosphatemia is associated with complications of bone mineral metabolism and mortality, and thus should be controlled in dialysis patients.^1^^,^ 2 Since conventional dialysis is insufficient to maintain a negative balance of phosphorus in most patients,^3^ the current treatment strategies are focused on improving the adequacy of dialysis whenever possible, provide nutritional guidance on adequate phosphorus intake, and prescription of phosphorus binders.^4^^,^ 5 Nonetheless, the prevalence of hyperphosphatemia is around 50% in developed countries. According to data from the Brazilian Society of Nephrology, the prevalence in Brazil is around 35% .^7^ Data from 2007 indicated 33% of patients in the Northern and Southern region and a 39% in the South region with phosphatemia above 5.5 mg/dL.^8^

In the last decade, even more striking differences have been observed between patients from the Northern state of Tocantins (TO) and the Southern state of Santa Catarina (SC), from a nation-wide institution. While the prevalence of hyperphosphatemia in patients from TO ranges from 20 to 30%, in SC it is between 45% and 55%. Since nutritional counseling, dialysis prescription, and access to phosphate binders are similar in the two state units, our hypothesis is that SC patients have higher consumption of phosphorus sources, both due to the typical dietary habits of the region and because of a higher income in SC, which allows access to a more diverse diet.

The dietary source of phosphorus can also have a significant influence on the phosphatemia of these patients. The organic phosphate from the natural sources has around 60% of bioavailability in a mixed diet, being lower in plant foods (such as legumes and nuts) and higher in animal sources (such as meats and dairy products). Inorganic phosphorus, which is added to foods and beverages during processing to increase shelf life and improve the flavor and color of products, is absorbed almost entirely in the intestine and can contribute in a very important way to the phosphatemia of these patients .^9^

Thus, due to the difficulty of evaluating the intake of phosphorus because of lack of information on the labels of processed foods, the objective of this study was to evaluate and compare the frequency of consumption of food and drink sources of phosphorus among patients from both states.

## METHODOLOGY

### Patients

This was a cross-sectional study that included a convenience sample of patients who were on a chronic hemodialysis program (three times a week, with sessions of four hours each) in three units of Santa Catarina (SC) and two units of Tocantins (TO) from the same institution. Inclusion criteria were: patients over 18 years of age who had been on hemodialysis for more than six months. Patients with impaired ability to understand the questionnaire were excluded. All patients had received tailored nutritional counseling and were followed monthly by a nutritionist. The protocol followed by the nutrition professionals recommends that, in the occurrence of hyperphosphatemia, a careful evaluation of the consumption of phosphorus dietary sources is performed. Nutritional counseling should prioritize the achievement or maintenance of adequate protein intake through sources with low phosphorus/protein ratio whenever possible. Patients are also advised to reduce consumption or avoid sources of inorganic phosphorus, such as ultra-processed foods and beverages. When indicated, adjustments in the phosphorus binder dosage are also suggested, according to phosphate level and the consumption of phosphorus sources.

### Food frequency questionnaire

During the months of January and February 2015, the recruited patients answered a food frequency questionnaire (FFQ) developed and applied by dietitians of the dialysis units. Due to the lack of a questionnaire previously validated for this population, a pre-standardized FFQ was used and applied during the usual unit routine rounds to investigate phosphorus consumption. The participating dietitians were encouraged to suggest the incorporation of other food items that are commonly reported by patients during clinical routine visits. The final version contained 33 items of organic phosphorus sources (naturally present in foods such as meat, dairy products, and legumes) and of inorganic phosphorus sources (as additives of processed foods and beverages, especially meats, artificial beverages, etc.). Participants were asked about seven possibilities of consumption frequency (1 = never, 2 = less than once a month, 3 = one to three times a month, 4 = once a week, 5 = two to four times a week, 6 = once per day and 7 = twice or more per day). For frequency analysis, the answers were grouped into three categories (answers 1 and 2, 3 to 5, and 6 and 7). In addition, a consumption score was calculated by adding the frequencies according to the number assigned to each answer possibility (1 to 7). The total score could vary from 33 to 231, for organic phosphorus sources from 19 to 133, and for inorganic phosphorus, from 14 to 98. Items containing both organic and inorganic phosphorus (such as processed meat) were classified as inorganic. The portion size of each item was also observed; however, due to the great variation of the phosphorus content in processed foods and the lack of information in labels, quantitative analysis was not performed.

### Other parameters

Participants were also questioned about schooling (years of formal study) and whether or not they were using any phosphate binder at the time. If so, the type and daily dose were recorded. Information on body weight, height, and time on dialysis were obtained from the electronic medical record. The laboratory tests were 6-month average serum phosphorus (due to intra-individual variability) and the last available serum parathyroid hormone (PTH) level.

### Statistical analysis

Statistical analysis was performed using SPSS software, version 21.0 for Windows (SPSS, Inc. Chicago, IL). Results are reported as mean and standard deviation or median and interquartile range, according to the distribution of variables determined by the Shapiro-Wilk test. For correlation analysis, the Pearson or Spearman test was used, when appropriate. For the comparison of the variables between groups, the Student's t-test was used for variables with normal distribution, or Mann-Whitney for variables with non-normal distribution. The chi-square test was used for the categorical variables. Statistical significance was considered for values of p <0.05.

## RESULTS

In total, 123 patients participated in the study, being the majority from SC (54%). As shown in Table 1, schooling was the demographic variable with the greatest discrepancy between the regions (higher in the South). SC patients had a higher mean BMI, phosphatemia, and prevalence of hyperphosphatemia; they also used phosphate binders more often and at higher doses.

**Table 1:** Characteristics of study patients

	Total (n=123)	SC (n= 66)	TO (n=57)	P (SC vs TO)
Men (%)	52	51	52	0.90
Age (years)	46.9 ± 15.7	47.4 ± 13.8	46.8 ± 17.7	0.82
Tine in HD (months)	48 (57-71)	48 (30-67)	38 (23-76)	0.45
Years of study	9 (4-11)	11 (7-11)	7 (4-10.5)	<0.001
BMI (kg/m^2^)	24.2 ± 4.7	25.1 ± 4.6	23.1 ± 4.7	0.02
Phosphorus (mg/dL)*	5.6 ± 1.6	6.2 ± 1.5	4.7 ± 1.3	<0.001
Hyperphosphatemia (%)	47	62	28	<0.001
PTH (pg/mL)	550 (275-968)	541 (260-938)	578 (312-1005)	0.92
Phosphate binder (%)	74	85	60	<0.001
Phosphate binder (nº pills/dia)	5 (3-6)	6 (5-6)	3 (2-4)	<0.001

* Six-month average. SC: Santa Catarina; TO: Tocantins; HD: hemodialysis; BMI: body mass index. PTH: parathyroid hormone.

The sources of phosphorus consumed most frequently in the total sample (at least once a day) were milk and beans (42% and 46%, respectively). Foods less frequently consumed (80% of participants reported consuming less than once a month or never) were sardines, seafood, processed chicken, beef liver, chicken giblets, peanuts, nuts, and beer.

A significant difference was found between the regions in 17 of the 33 items analyzed (Table 2). Patients from TO consumed beef and beans more frequently, while those from SC reported higher consumption of pork, dairy products, and wieners, among others.

**Table 2:** Consumption frequency of food items in the FFQ significantely differente between the states.

Food item	SC			TO			P
	I (%)	II (%)	III (%)	I (%)	II (%)	III (%)	
Meet	3	86	11	9	63	28	0.01
Pork	50	50	0	70	30	0	0.02
Sardine/tuna	73	27	0	91	9	0	0.007
Sea food	86	14	0	100	0	0	0.003
Ham	35	54	11	39	61	0	0.04
Mortadella/salami	65	29	6	89	11	0	0.004
Weiner	58	42	0	89	9	2	<0.001
Hamburger (processed)	71	27	2	91	9	0	0.02
Milk	17	24	59	17	60	23	<0.001
Cheese	15	67	18	37	58	5	0.006
Pizza	47	53	0	84	14	2	<0.001
Chocolate/chocolate powder	53	39	8	74	26	0	0.02
Milk sweets	50	45	5	77	21	2	0.008
Peanuts/sweets with peanuts	46	23	1	93	7	0	0.03
Nuts	86	14	0	100	0	0	0.004
Beans	9	59	32	3	33	63	0.002
Beer	80	20	0	95	5	0	0.02

SC: Santa Catarina; TO: Tocantins; I: never or less than once a month; II: once to 3x per month, once to 3x per week; III: once to 3x per day.

The phosphorus consumption score of the total sample was 74±13 points, with 49±7 points from organic sources and 25±7 from the inorganic sources. Patients from SC had the highest total score (78±11 vs 70±14, p = 0.002) and organic sources score (51±7 vs 46±7, p <0.001). There was no difference in the score for inorganic sources (35±19 vs 30±22, p = 0.14).

Significant correlations between consumption frequency scores and biochemical and demographic parameters are presented in Table 3. The three scores correlated inversely with age and directly with schooling. The only factor that significantly correlated with phosphatemia was the frequency of consumption of inorganic phosphorus sources.

**Table 3:** Characteristics of study patients.

Score	Parameter	R	P
Total	Age	-0.39	<0.001
	Schooling	0.26	0.004
Organic	Age	-0.27	0.003
	Inorganic score	0.50	<0.001
	Schooling	0.24	0.008
Inorganic	Age	-0.41	<0.001
	Schooling	0.21	0.02
	Phosphatemia	0.20	0.03

## DISCUSSION

The present study showed that patients on dialysis therapy in SC consumed more frequently foods that contain phosphorus than patients in TO, and this may be related to the worse control of phosphatemia found in the sample from this region.

The difference in consumption frequencies between regions is in line with national data provided by the Family Budget Survey (POF 2008-2009), which quantitatively measured the annual family (per capita) acquisition of various foods and beverages in all regions of the country. As observed in that survey, the population of the Northern region consumes more legumes (10.2 vs 6.8 kg) and beef than the Southern (23.7 vs 21.8 kg). The Southern region consumes almost three times more pork (including processed) (5.0 vs 1.8 kg) and dairy products (67.4 vs 24 kg). The survey also reported higher intake of chocolate, milk-based sweets, and nuts among the population of the Southern region.^10^

Food patterns differ among populations due to several factors, such as regional availability, culture, family tradition, religion, climate, among others. More than 2,000 kilometers separate Santa Catarina from Tocantins. While the territory of Santa Catarina was strongly influenced by European colonization, especially since the 19th century, and the state is located in a subtropical climate, the newly created (1988) state of Tocantins underwent a recent miscegenation of local residents and migrants from other parts of the country and the State has a dry tropical climate.

Food preferences are also driven by family income, which may favor or hinder access to different foods and beverages. Although we did not investigate this variable, the percentage of patients undergoing hemodialysis in 2017 in the institution with a per capita family income below the minimum wage was 33% in SC units and 68% in TO units. However, the important difference in schooling (which directly influences family income)^10^ found in our study indicates that participants treated in the South had higher income than those in the North. In addition, a significant and direct correlation was found between schooling and the three consumption frequency scores, showing that patients with higher schooling consumed a greater variety of phosphorus sources.

The relationship between socioeconomic status and phosphatemia was well explored in an American study that included almost 3,000 patients on hemodialysis. In that study, patients with lower socioeconomic status or who were unemployed had higher phosphatemia than those who were at higher socioeconomic levels or who were working. Although they did not investigate intake patterns, the authors believe that this finding is a consequence of the possible higher consumption of processed and low-cost, phosphorus-rich foods.

The inverse relationship between consumption scores and age may have been influenced by both the schooling factor (lower among the elderly) and the natural decrease in metabolism and appetite related to age advancement.^12^

The only score that correlated with phosphatemia was the frequency of consumption of inorganic phosphorus. As mentioned earlier, the frequent intake of foods rich in phosphorus additives can significantly influence phosphatemia because this type of phosphate is highly absorbed by the body. In fact, a Brazilian study found very high phosphorus concentrations in the industrialized products commonly consumed by dialysis patients in the Southeastern region.^13^ In addition, a randomized controlled trial, also conducted in Brazil with hemodialysis patients who received nutritional counseling for avoiding processed foods and giving preference to *in natura* products, showed a significant reduction of phosphatemia after 90 days, with the maintenance of protein intake.^14^

An American study also yielded positive results with an educational intervention focused on reducing the consumption of food additive sources. Among the actions, the patients in the study group were given magnifying glasses to help read the nutritional information label of processed foods and instructed to avoid those containing added phosphate.^15^ As in our country, in the USA, the information about the phosphorus content on food and beverage labels is not required by law.

In previous studies conducted in SC units, our group also showed that nutritional counseling led to a decrease in phosphatemia.^16^ However, although patients had a good knowledge about the treatment of hyperphosphatemia, 87% stated that they consume more phosphorus than recommended and/or did not follow the recommended prescription of phosphorus binders.^17^ In a more recent study, in which we investigated the patients' perception of adherence to dietary restrictions, it was found that limiting the consumption of phosphorus was difficult for most patients, justifying that they enjoyed foods and drinks that are sources of phosphorus.^18^

As limitations, we highlight the use of a non-validated FFQ, which may have omitted food items that were important for the study. In addition, the relatively small sample from each State and the low average age may have limited the representativeness of the results.

## CONCLUSION

This study showed that there are important differences in the consumption of phosphorus sources among Southern and Northern patients, which is in agreement with what is observed in the general population. These findings emphasize that nutritional counseling, as well as the development of educational material, must consider the cultural and socioeconomic factors of the place where strategies will be applied. Although we cannot state that there is a greater absolute intake of phosphorus by patients in the South, the higher consumption of several foods found in SC may be closely related to the higher concentrations of serum phosphorus and the prevalence of hyperphosphatemia in this population.
