# Erratum: Differences in phosphatemia and frequency of consumption of dietary sources of phosphorus in hemodialysis patients in southern and northern Brazil

**DOI:** 10.1590/2175-8239-JBN-2018-0063er

**Published:** 2018-12-10

In the article "Differences in phosphatemia and frequency of consumption of dietary sources of phosphorus in hemodialysis patients in southern and northern Brazil" with DOI code number http://dx.doi.org/10.1590/2175-8239-jbn-2018-0063 published at Brazilian Journal of Nephrology in 2018:

Where it was written:

**Introduction:** Hyperphosphatemia is associated with unfavorable outcomes, and the percentage of patients presenting with this condition in hemodialysis (HD) in kidney foundation units in the state of Santa Catarina (SC) is historically higher than that of patients in the state of Tocantins (TO). **Objective:** To assess the frequency of consumption of the main dietary sources of phosphorus and to compare them between the two states. **Methodology:** A cross-sectional study was carried out involving 123 patients, 66 of SC and 57 of TO: 52% were men, average age was 46.9 ± 15.7 years, and mean HD time 48 (57-71) months. A food frequency questionnaire (FFQ) with 33 items that are dietary sources of phosphorus was applied. A consumption score was calculated for sources of organic, inorganic, and total phosphorus, and the six-month average of phosphatemia was obtained. **Results:** The mean phosphatemia of SC patients was higher (6.2 ± 1.5 vs. 4.7 ± 1.3 mg/dL, *p* <0001) than TO patients, as well as the prevalence of hyperphosphatemia (62% vs. 28%; *p* <10001). In the total sample, the foods most frequently consumed were milk and beans. Comparing the frequency of consumption between the two states, a significant difference was found in 17 items. In TO, beef and beans were the foods most frequently consumed, and in SC, fourteen other items of the FFQ (pork, sausages, dairy products, etc.) were the most frequently consumed. Phosphatemia correlated with the frequency of consumption of inorganic phosphorus sources. **Conclusion:** the frequency of consumption of several items was different between the states, and this explains the differences in phosphatemia between the two regions.

Should read:

**Introduction:** Hyperphosphatemia is associated with poor outcomes and the percentage of patients with this condition in hemodialysis (HD) units from the same institution in Southern Brazil (Santa Catarina State (SC)) is historically higher than in patients in Northern Brazil (Tocantins State (TO). **Objective:** To know the frequency of consumption of the main dietary sources of phosphorus and to compare it between the two populations. **Methodology:** Cross-sectional study. We included 123 patients, 66 from SC and 57 from TO (52% men, age = 46.9±15.7 years, HD vintage = 48(57-71) months). A food frequency questionnaire (FFQ) with 33 dietary sources of phosphorus was applied. A consumption score was calculated for sources of organic, inorganic and total phosphorus. **Results:** The mean 6 months phosphatemia of SC patients was higher (6.2 ± 1.5 vs. 4.7 ± 1.3 mg/dl, *p* <0001), as well as the prevalence of hyperphosphatemia (62% vs. 28%). In the total sample, food items most frequently consumed were milk and beans. When comparing the frequency of consumption of the FFQ items between the two populations, a significant difference was found in 17 items. In TO, there was a higher frequency of consumption of beef and beans, while in SC of other fourteen items of the FFQ (pork, sausages, dairy products, etc.). Phosphatemia correlated with the frequency of consumption of inorganic phosphorus sources score. **Conclusion:** the frequency of consumption of several items was different between the states and this finding may be one of the reasons explaining the disparities in phosphatemia observed in these two regions.

